# How Can Genetic Studies Help Us to Understand Links Between Birth Weight and Type 2 Diabetes?

**DOI:** 10.1007/s11892-017-0852-9

**Published:** 2017-03-14

**Authors:** Robin N. Beaumont, Momoko Horikoshi, Mark I. McCarthy, Rachel M. Freathy

**Affiliations:** 1 Institute of Biomedical and Clinical Science, University of Exeter Medical School, University of Exeter, RILD Building, Royal Devon and Exeter Hospital, Barrack Road, Exeter, EX2 5DW UK; 2 Wellcome Trust Centre for Human Genetics, University of Oxford, Roosevelt Dr., Oxford, OX3 7BN UK; 3 Oxford Centre for Diabetes, Endocrinology and Metabolism, University of Oxford, Oxford, UK; 4 Oxford National Institute for Health Research (NIHR) Biomedical Research Centre, Churchill Hospital, Old Road, Headington, Oxford, OX3 7IL UK; 5 Medical Research Council Integrative Epidemiology Unit, University of Bristol, Bristol, UK

**Keywords:** Birth weight, Fetal, Genetics, Genome-wide association study, Maternal, Type 2 diabetes

## Abstract

**Purpose of Review:**

In observational epidemiology, both low and high birth weights are associated with later type 2 diabetes. The mechanisms underlying the associations are poorly understood. We review evidence for the roles of genetic and non-genetic factors linking both sides of the birth weight distribution to risk of type 2 diabetes, focusing on contributions made by the most recent genome-wide association studies (GWAS) of birth weight.

**Recent Findings:**

There are now nine genetic loci robustly implicated in both fetal growth and type 2 diabetes. At many of these, the same alleles are associated both with a higher risk of type 2 diabetes and a lower birth weight. This supports the Fetal Insulin Hypothesis and reflects a general pattern for type 2 diabetes susceptibility alleles: genome-wide, there is an inverse genetic correlation with birth weight, and initial estimates suggest genetic factors explain a large part of the covariance between the two traits. However, the associations at individual loci show heterogeneity; some fetal risk alleles are associated with higher birth weight. For most of these, the association reflects their correlation with the maternal risk allele which raises maternal glucose, thus increasing fetal insulin-mediated growth.

**Summary:**

GWAS have improved our understanding of the mechanisms underlying associations between type 2 diabetes and birth weight but questions remain about the relative importance of genetic versus non-genetic factors and of maternal versus fetal genotypes. To answer these questions, future work will require well-powered analyses of parents and offspring.

## Introduction

The rising global prevalence of type 2 diabetes presents a major challenge for human health. Both environmental and genetic factors contribute to this complex disease. The roles of obesogenic diet and low physical activity as major adult risk factors are well established. Moreover, in any given population, there are individuals whose profile of genetic variation confers greater susceptibility, for example by predisposing to pancreatic beta cell dysfunction. Most of the genetic variance in type 2 diabetes susceptibility is attributable to variants of common frequency in populations [1•].

**Fig. 1:**
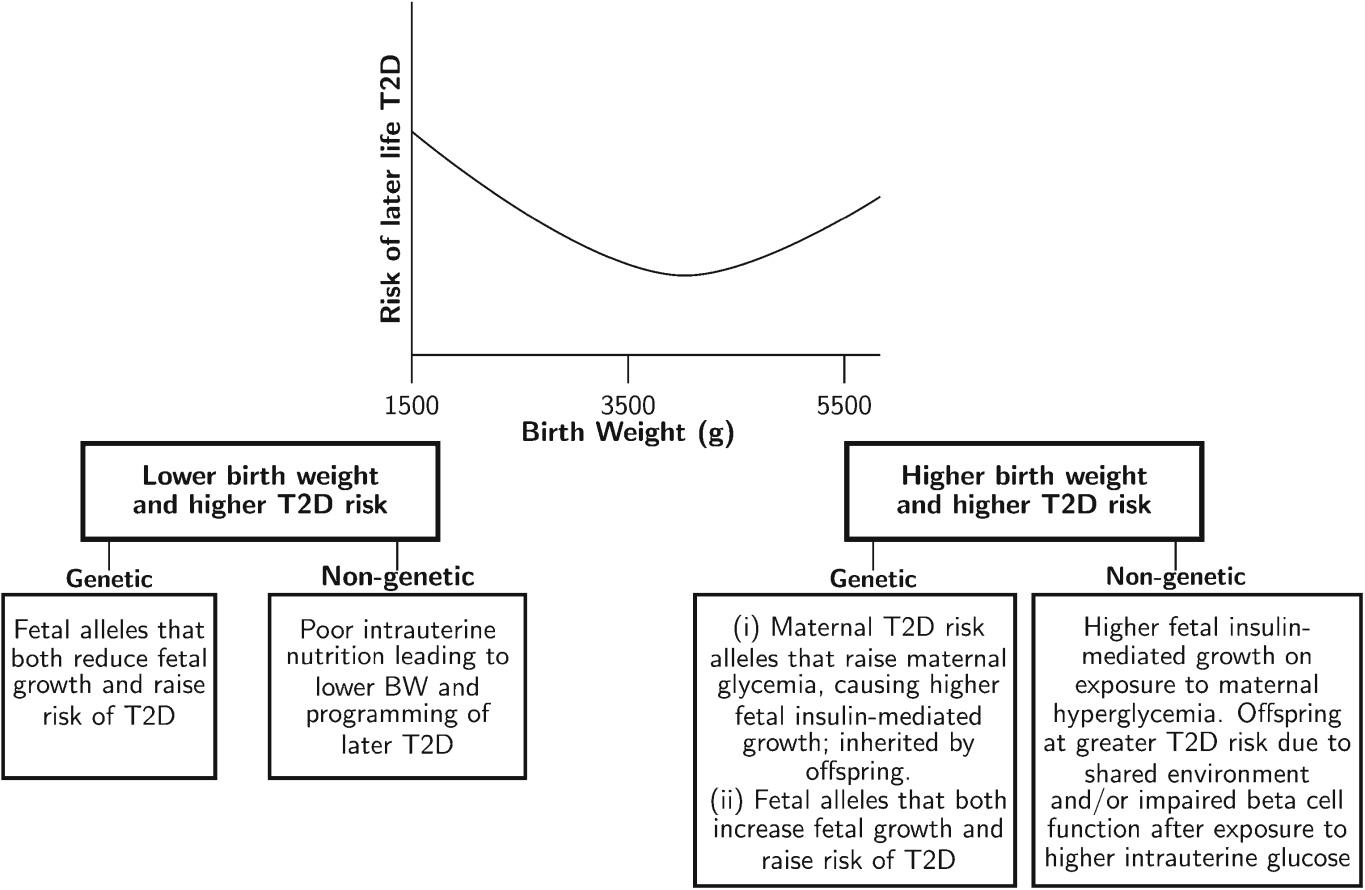
Genetic and non-genetic factors underlying the observational association between birth weight (BW) and type 2 diabetes (T2D). The plot is a schematic representation of the U-shaped relationship resulting from meta-analysis of multiple observational studies [5]

An additional associated factor, which has attracted much attention during the past 25 years, is birth weight. Observational studies have consistently reported associations between type 2 diabetes risk in adulthood and either low or high birth weight [2–4]. Taken together, these studies support a U-shaped relationship (Fig. 1), with those individuals at both extremes of the distribution at greater risk [5]. In this review, we will consider both sides of the “U” separately and examine how genetic studies have contributed to our understanding of the underlying mechanisms. An overview of the mechanisms involved is presented in Fig. 1.

## Evidence that Non-genetic Factors Underlie the Association Between Lower Birth Weight and Type 2 Diabetes

The observation that individuals born with lower birth weights were more likely to have impaired glucose tolerance in middle-age was first demonstrated 25 years ago [3], and has since been replicated in multiple geographically and ethnically diverse populations. These observations led to the hypothesis that adverse conditions in utero, for example poor nutrition, would result not only in reduced fetal growth but also in physiological adaptations that increase susceptibility to poor adult health [6, 7]. Evidence to support this Developmental Origins of Health and Disease (DOHaD) hypothesis is reviewed comprehensively in [8], but there are two key lines of evidence from human studies that provide support for non-genetic mechanisms linking lower birth weight to later life type 2 diabetes risk. First, data from studies of famines (e.g., in the Netherlands, 1944–5 or China, 1959–61) enable inference to be made about the long-term effects of exposure to famine during the gestation period alone. Such studies have shown that babies born to mothers who were pregnant during famine had reduced glucose tolerance later in life compared with those who were in utero outside of famine periods [9, 10]. Second, in a study of monozygotic twins discordant for type 2 diabetes, the affected twins had lower birth weights, on average, than the unaffected twins, despite sharing the same genetic predisposition [11].

## Evidence that Genetic Factors Underlie the Association Between Lower Birth Weight and Type 2 Diabetes

The principle that genetic factors could be important was established in studies of monogenic diabetes. In 1998, Hattersley et al. [12] demonstrated that inheriting mutations in the glucokinase gene (*GCK*), which reduce glucose sensing by the pancreas, cause both a reduction in insulin-mediated fetal growth and hyperglycemia after birth. Based on this and other rare, monogenic examples of low birth weight with either impaired insulin secretion or greater insulin resistance, the Fetal Insulin Hypothesis was proposed [13]; alleles that predispose to reduced insulin secretion or raised insulin resistance will both reduce insulin-mediated growth in utero and increase type 2 diabetes risk in adulthood.

**Fig. 2:**
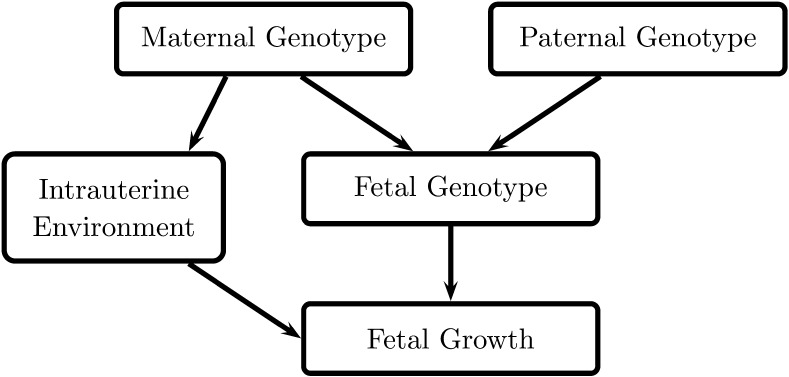
Diagram showing direct and indirect effects of parental genotype on fetal growth. The mother’s genotype may influence birth weight directly, by being inherited by the fetus, or indirectly via a primary effect on the intrauterine environment (e.g., glucose availability). The father’s genotype cannot influence the intrauterine environment in this way, so is expected only to influence birth weight directly, once inherited

Evidence to support a role for polygenic effects in the link between type 2 diabetes risk and birth weight has come from epidemiological studies, e.g., [2, 14], which showed that paternal diabetes status is associated with lower birth weight, while maternal diabetes status is associated with higher birth weight. Since a father can pass on only genes to the offspring, and not influence the intrauterine environment in the same way as the mother (Fig. 2), these observations suggest that inheriting diabetes susceptibility variants reduces fetal growth.

**Fig. 3:**
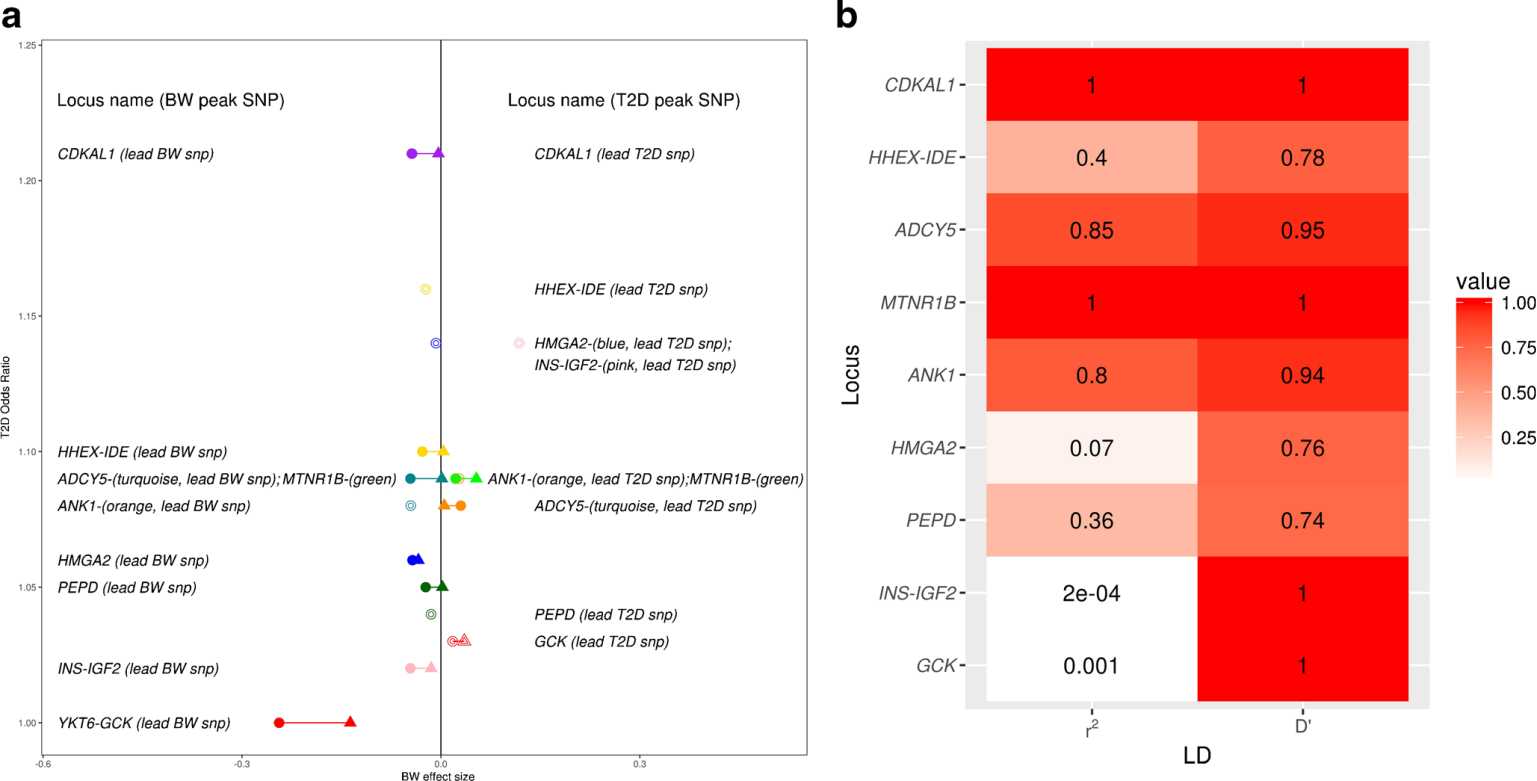
Associations of SNPs at nine loci identified in GWAS with birth weight and type 2 diabetes. **a** Type 2 diabetes (T2D) odds ratio [1, 21] (*y*-axis) plotted against birth weight (BW) effect size in SD units (*x*-axis) of SNPs at nine loci implicated in GWAS of both traits. *Solid circles* indicate fetal genotype associations for peak (i.e., most strongly associated) SNPs identified in fetal GWAS of birth weight [20••], while *solid triangles* indicate the corresponding maternal genotype associations at the same SNPs [20••]. *Open circles* indicate fetal genotype associations for peak SNPs identified in GWAS of T2D [1, 21–26]. The *open triangles* indicate corresponding maternal genotype associations (available only for *GCK* [27] and *MTNR1B* [20••, 27]) at the same (or perfectly-correlated) SNPs. **b** Pairwise linkage disequilibrium (LD) estimates (in European samples) between peak birth weight SNPs [20••] and peak T2D SNPs [1, 21–26] at the nine loci implicated in both traits. An *r* ^2^ close to 1 implies that the peak SNPs for both traits provides almost identical information. For example, at *CDKAL1, MTNR1B, ADCY5*, and *ANK1*, the birth weight and type 2 diabetes peak SNPs are very closely correlated, so the birth weight and T2D association signals can be assumed to be tagging the same causal variant (also likely the case for *HHEX-IDE* and *PEPD*, where *r* ^2^ ≈ 0.4). Conversely, there is a low correlation between the BW and T2D peak SNPs at *INS-IGF2, GCK*, and *HMGA2*), raising the possibility that they could be tagging different association signals within the same region of LD (indicated by D’ of 1). In particular, the very different birth weight and T2D associations of the two *GCK* SNPs support this—the birth weight peak SNP from the fetal GWAS at this locus (also encompassing *YKT6*) is of low minor allele frequency (European MAF <1%) and shows no evidence of association with T2D, whereas the T2D peak SNP is common (European MAF = 18%), with some evidence of association between the maternal risk allele and higher birth weight. The latter is consistent with a primary effect on maternal hyperglycemia

During the past 10 years, the identification of type 2 diabetes susceptibility variants through genome-wide association studies (GWAS), coupled with increasingly well-powered genetic studies of birth weight, enabled the first evidence to emerge that multiple common genetic variants could contribute both to variation in birth weight and type 2 diabetes risk. Evidence from candidate gene studies [15–17], and the first GWAS [18, 19] of birth weight suggested that the type 2 diabetes risk alleles at the *CDKAL1*, *ADCY5*, and *HHEX*-*IDE* loci were also associated with lower birth weight. It is likely that these associations were mediated through their effects on insulin secretion. The most recent GWAS of birth weight [20••], in 153,781 individuals, identified 60 loci at genome-wide significance (here defined as *P* < 5 × 10^−8^), nine of which had also been associated with type 2 diabetes (Fig. 3). Most of the type 2 diabetes risk alleles at these nine loci showed associations with lower birth weight. However, risk alleles at *MTNR1B*, *GCK*, and *ANK1* were associated with higher birth weight.

## GWAS Enable Genome-Wide Assessments of the Genetic Link Between Type 2 Diabetes and Birth Weight

Genetic variation at the 60 birth weight loci and approximately 100 type 2 diabetes susceptibility loci explain approximately 2 and 5–10% of the variation in these traits, respectively. Assays of common fetal genetic variation across the whole genome have been estimated collectively to explain 15% of the variance in birth weight and at least 49% of liability-scale variance in type 2 diabetes risk [20, 22]. However, it is not clear what percentage of the covariation between type 2 diabetes and birth weight is genetic.

To estimate the overall genetic correlation between type 2 diabetes and birth weight, genome-wide summary statistics may be combined using a technique known as LD score regression [28•]. This method estimates the shared genetic component between the two traits and distinguishes it from confounding due to environmental exposures. LD score regression supports an inverse genetic correlation between birth weight and type 2 diabetes [20]. In other words, type 2 diabetes risk alleles across the genome also tend to be associated with lower birth weight. But how much of the covariation, i.e., the tendency of birth weight and type 2 diabetes risk to track together, is due to genetics? This question can be tackled using individual-level data in a mixed model analysis, which enables partitioning of the covariance between the two traits into genetic and residual components [29]. Initial estimates using UK Biobank data suggested that a large proportion (96%) of the covariance was due to shared genetic factors [20], though the confidence intervals around this estimate were wide.

While genome-wide correlation and covariance estimates lend support for a substantial role of common genetic effects contributing to the low birth weight–type 2 diabetes association, it is important to consider their limitations. The methods used assume a linear relationship between the two phenotypes, which is not strictly applicable to the overall non-linear relationship that has been observed between type 2 diabetes risk and birth weight.

The fact that mother and child share 50% of their genes also means that care must be taken in interpreting estimates of genetic covariance between birth weight and type 2 diabetes. The correlation (r ≈ 0.5) between maternal and fetal genotype means that shared genetic effects are not necessarily directly attributable to the fetus. Fetal genetic associations may in fact indicate effects of the intrauterine environment, influenced by maternal genotype (see Fig. 2). A method for separating these effects in relation to a single trait has been developed by Eaves et al. [30•]. The model, called “m-GCTA”, uses both maternal and fetal genotype to estimate the relative contribution of maternal vs. fetal genotype to a trait. Applying this to data from the Avon Longitudinal Study of Parents and Children (ALSPAC) showed that the maternal genotype contributes relatively little compared with fetal genotype to variance in birth weight [20]. It may follow that fetal genotype contributes to the majority of the covariance between birth weight and type 2 diabetes, but it has not yet been possible to apply this method to covariance. Methods that account both for non-linear relationships and maternal vs. fetal effects will need to be applied to large studies of mothers and babies to explore these genome-wide genetic effects further.

In summary, there is evidence that both genetic and non-genetic factors underlie the observed association between lower birth weight and higher risk of type 2 diabetes (Fig. 1, left side), and initial estimates using GWAS data suggest that genetic factors play the major role. The associations at three of the nine loci now robustly implicated in both traits (Fig. 3) indicate, however, that some type 2 diabetes risk alleles are associated with higher, not lower, birth weight, and therefore may contribute to the other side of the U-shaped curve.

## Evidence that Non-genetic Factors Underlie the Association Between Higher Birth Weight and Type 2 Diabetes

The association between higher birth weight and type 2 diabetes is more apparent in populations with high type 2 diabetes prevalence, for example, the Pima Indian population of Arizona. In this population, children born to mothers who showed signs of diabetes in pregnancy had higher birth weights and higher rates of type 2 diabetes later in life than those born to non-diabetic mothers [31]. These higher rates of diabetes might reflect a stronger genetic predisposition, but it was also observed that the offspring of women who developed diabetes before pregnancy had a higher prevalence of type 2 diabetes than in those whose mothers developed diabetes after pregnancy. Exposure to maternal hyperglycemia in utero results in increased fetal growth as a result of higher fetal insulin secretion, while shared environmental factors post-birth will contribute to a higher risk of type 2 diabetes in the offspring. But the difference between the offspring of prediabetic vs. diabetic mothers suggests that intrauterine-hyperglycemia exposure additionally contributes to a higher risk of type 2 diabetes in later life, likely through an adverse effect on offspring beta cell function (Fig. 1, right side).

Studies of monogenic diabetes and type 1 diabetes have provided additional evidence in support of such intrauterine effects; an 8-year earlier age of diagnosis was observed for individuals inheriting a diabetes-causing mutation in *HNF1A* if this was inherited from their mother (who had diabetes while pregnant), than if inherited from their father [32]; and in a study of the offspring of parents with type 1 diabetes, those with affected mothers had poorer glucose tolerance and beta cell function than those with affected fathers [33].

## Evidence that Genetic Factors Underlie the Association Between Higher Birth Weight and Type 2 Diabetes

As illustrated in Fig. 3, there are examples of individual loci that show positive associations with both birth weight and type 2 diabetes risk [20••]. At the *MTNR1B* locus, both the fetal and maternal risk allele at SNP rs10830963 have been identified in GWAS as associated with higher birth weight [20••, 27]. The maternal effect size is larger than the fetal effect size at this SNP, and it is likely that the association observed with the fetal risk allele is merely due to correlation with the allele in the mother. The risk allele at *MTNR1B* has one of the largest effects of common variants known to influence fasting glucose levels [34], thus the likely mechanism underlying the birth weight association is of higher fetal insulin secretion in response to higher maternal glucose levels. Inheritance of the risk allele by the offspring would then explain the link with the later higher risk of type 2 diabetes.

Similar patterns of association have been observed between maternal type 2 diabetes risk alleles and higher offspring birth weight at the *GCK* and *TCF7L2* loci [35–37], both of which are known to influence fasting glucose levels in adults without diabetes [23]. However, larger sample sizes will be required to confirm the birth weight associations with genome-wide levels of statistical support. The common variant at *GCK* is likely to represent an association signal distinct from the nearby low frequency variant that has been associated with birth weight when present in the fetus [20] (see Fig. 3).

Finally, variation at one locus, *ANK1*, shows association with both higher birth weight and higher risk of type 2 diabetes [20], but unlike *MTNR1B*, this association does not appear to be driven by the maternal genotype, raising the possibility of a genetic mechanism contributing to the observational association between higher birth weight and type 2 diabetes that operate independently of maternal hyperglycemia.

## The Relative Effects on Birth Weight of Genetic Variants Associated with Type 2 Diabetes Give Clues to the Timing of their Physiological Effects

The variety of associations observed between known type 2 diabetes variants and birth weight enable us to make a number of inferences about their mechanisms of action. Of the nine loci in Fig. 3, those with the largest birth weight-lowering effects (*CDKAL1*, *ADCY5*, *HHEX-IDE*) also have relatively large effects on type 2 diabetes risk. These three loci are all classified as associated with reduced beta cell function [38], and their associations with birth weight suggest that they may start to influence insulin secretion during early growth. In notable contrast, the fetal risk allele at the common variant locus with the largest effect on type 2 diabetes risk, *TCF7L2*, is not associated with lower birth weight [20], strongly suggesting it confers little or no insulin secretory defect in fetal life.

Alleles showing the largest effects on raising birth weight through the maternal genotype (*MTNR1B*, *GCK*) are known to have relatively large effects on fasting glucose and more moderate effects on type 2 diabetes risk. The balance of maternal birth weight-raising vs. fetal birth weight-lowering effects might be viewed as a bioassay on the life-course timing of physiological effects on beta-cell function. For example, a larger maternal than fetal effect suggests the allelic effect is less prominent in early life than in later life. To disentangle the contribution of the mother’s genotype from that of the offspring, and to estimate accurately the maternal and fetal effects at individual loci will require extremely large samples of mother-child pairs.

## Future Studies will Require the Analysis of both Parent and Offspring Genotype Data

GWAS of birth weight have to date focussed solely on either the fetal or the maternal genotype. This is in part due to the limited number of studies with comprehensive phenotype and genotype data on both offspring and parents. To better understand the associations between birth weight and type 2 diabetes and the relative effects of maternal and fetal genotypes on early development, it will be important to include both maternal and fetal data in the same analyses.

One approach to disentangling maternal from fetal effects is to define and use the maternally transmitted, non-transmitted and paternally inherited alleles. In the absence of assortative mating, the maternal non-transmitted and paternally inherited alleles should be uncorrelated with the maternal-transmitted allele, therefore allowing differentiation between maternal and fetal effects. This has been done successfully using a genetic score of height-raising alleles in relation to birth weight and length, showing the predominant importance of transmitted alleles [39•]. In future, well-powered studies, this method has the potential to clarify the role of maternal vs. fetal effects on birth weight variation and its association with type 2 diabetes.

Type 2 diabetes loci have been identified that express effects on disease risk in a parent-of-origin specific manner [40]. To date, no parental origin-specific effects of common genetic variants have been demonstrated for birth weight. However, the latest fetal GWAS of birth weight observed enrichment of associations in imprinted genes and three loci at, or close to, genome-wide significance encompassed imprinted regions of the genome (near *INS-IGF2*, *RB1*, and *DLK1*) [20••]. Future well-powered studies of parent-offspring trios or mother-child pairs will enable detection of any underlying parent-of-origin effects.

## Conclusions

The U-shaped association between type 2 diabetes and birth weight is underpinned by multiple genetic and non-genetic factors. Recent GWAS data have greatly improved our understanding of the genetic factors contributing to the two traits, providing examples of individual loci that explain both positive and inverse covariation between them. Moreover, genome-wide genotype data in individuals with life-course phenotype data has enabled initial estimates of the overall role of genetic factors, suggesting they may play a major role in the association between type 2 diabetes and lower birth weight. Many questions remain unanswered, however, such as the relative importance of maternal and fetal genetic effects both genome-wide and at individual loci, and it is likely that great future gains will be made from well-powered analyses of parents and offspring.

## References

[1] Fuchsberger C, Flannick J, Teslovich TM, Mahajan A, Agarwala V, Gaulton KJ (2016). The genetic architecture of type 2 diabetes. Nature.

[2] Tyrrell JS, Yaghootkar H, Freathy RM, Hattersley AT, Frayling TM (2013). Parental diabetes and birthweight in 236 030 individuals in the UK biobank study. Int J Epidemiol.

[3] Hales CN, Barker DJ, Clark PM, Cox LJ, Fall C, Osmond C (1991). Fetal and infant growth and impaired glucose tolerance at age 64. BMJ.

[4] Dabelea D, Pettitt DJ, Hanson RL, Imperatore G, Bennett PH, Knowler WC (1999). Birth weight, type 2 diabetes, and insulin resistance in Pima Indian children and young adults. Diabetes Care.

[5] Harder T, Rodekamp E, Schellong K, Dudenhausen JW, Plagemann A (2007). Birth weight and subsequent risk of type 2 diabetes: a meta-analysis. Am J Epidemiol.

[6] Barker DJ, Hales CN, Fall CH, Osmond C, Phipps K, Clark PM (1993). Type 2 (non-insulin-dependent) diabetes mellitus, hypertension and hyperlipidaemia (syndrome X): relation to reduced fetal growth. Diabetologia.

[7] Godfrey KM, Barker DJ (2000). Fetal nutrition and adult disease. Am J Clin Nutr.

[8] Martin-Gronert MS, Ozanne SE (2012). Mechanisms underlying the developmental origins of disease. Rev Endocr Metab Disord.

[9] de Rooij SR, Painter RC, Roseboom TJ, Phillips DI, Osmond C, Barker DJ (2006). Glucose tolerance at age 58 and the decline of glucose tolerance in comparison with age 50 in people prenatally exposed to the Dutch famine. Diabetologia.

[10] Li Y, He Y, Qi L, Jaddoe VW, Feskens EJ, Yang X (2010). Exposure to the Chinese famine in early life and the risk of hyperglycemia and type 2 diabetes in adulthood. Diabetes.

[11] Poulsen P, Vaag AA, Kyvik KO, Møller Jensen D, Beck-Nielsen H (1997). Low birth weight is associated with NIDDM in discordant monozygotic and dizygotic twin pairs. Diabetologia.

[12] Hattersley AT, Beards F, Ballantyne E, Appleton M, Harvey R, Ellard S (1998). Mutations in the glucokinase Gene of the fetus result in reduced birth weight. Nat Genet.

[13] Hattersley AT, Tooke JE (1999). The fetal insulin hypothesis: an alternative explanation of the association of low birthweight with diabetes and vascular disease. Lancet.

[14] Lindsay RS, Dabelea D, Roumain J, Hanson RL, Bennett PH, Knowler WC (2000). Type 2 diabetes and low birth weight: the role of paternal inheritance in the association of low birth weight and diabetes. Diabetes.

[15] Freathy RM, Bennett AJ, Ring SM, Shields B, Groves CJ, Timpson NJ (2009). Type 2 diabetes risk alleles are associated with reduced size at birth. Diabetes.

[16] Andersson C, Olesen JB, Hansen PR, Weeke P, Norgaard ML, Jørgensen CH (2010). Metformin treatment is associated with a low risk of mortality in diabetic patients with heart failure: a retrospective nationwide cohort study. Diabetologia.

[17] Zhao J, Li M, Bradfield JP, Wang K, Zhang H, Sleiman P (2009). Examination of type 2 diabetes loci implicates *CDKAL1* as a birth weight Gene. Diabetes.

[18] Freathy RM, Mook-Kanamori DO, Sovio U, Prokopenko I, Timpson NJ, Berry DJ (2010). Variants in *ADCY5* and near *CCNL1* are associated with fetal growth and birth weight. Nat Genet.

[19] Horikoshi M, Yaghootkar H, Mook-Kanamori DO, Sovio U, Taal HR, Hennig BJ (2013). New loci associated with birth weight identify genetic links between intrauterine growth and adult height and metabolism. Nat Genet.

[20] Horikoshi M, Beaumont RN, Day FR, Warrington NM, Kooijman MN, Fernandez-Tajes J (2016). Genome-wide associations for birth weight and correlations with adult disease. Nature.

[21] Ng MC, Shriner D, Chen BH, Li J, Chen WM, Guo X (2014). Meta-analysis of genome-wide association studies in African Americans provides insights into the genetic architecture of type 2 diabetes. PLoS Genet.

[22] Morris AP, Voight BF, Teslovich TM, Ferreira T, Segrè AV, Steinthorsdottir V (2012). Large-scale association analysis provides insights into the genetic architecture and pathophysiology of type 2 diabetes. Nat Genet.

[23] Dupuis J, Langenberg C, Prokopenko I, Saxena R, Soranzo N, Jackson AU (2010). New genetic loci implicated in fasting glucose homeostasis and their impact on type 2 diabetes risk. Nat Genet.

[24] Mahajan A, Go MJ, Zhang W, Below JE, Gaulton KJ, Ferreira T (2014). Genome-wide trans-ancestry meta-analysis provides insight into the genetic architecture of type 2 diabetes susceptibility. Nat Genet.

[25] Imamura M, Takahashi A, Yamauchi T, Hara K, Yasuda K, Grarup N (2016). Genome-wide association studies in the Japanese population identify seven novel loci for type 2 diabetes. Nat Comm.

[26] Cho YS, Chen CH, Hu C, Long J, Ong RT, Sim X (2011). Meta-analysis of genome-wide association studies identifies eight new loci for type 2 diabetes in east Asians. Nat Genet.

[27] Feenstra B, Beaumont RN, Cavadino A, Tyrrell J, McMahon G, Nodzenski M (2016). Maternal genome-wide association study identifies a fasting glucose variant associated with offspring birth weight. BiorXiv.

[28] Bulik-Sullivan B, Finucane HK, Anttila V, Gusev A, Day FR, Loh PR (2015). An atlas of genetic correlations across human diseases and traits. Nat Genet.

[29] Loh PR, Bhatia G, Gusev A, Finucane HK, Bulik-Sullivan BK, Pollack SJ (2015). Contrasting genetic architectures of schizophrenia and other complex diseases using fast variance-components analysis. Nat Genet.

[30] Eaves LJ, Pourcain BS, Smith GD, York TP, Evans DM (2014). Resolving the effects of maternal and offspring genotype on dyadic outcomes in genome wide complex trait analysis (“M-GCTA”). Behav Genet.

[31] Pettitt DJ, Aleck KA, Baird HR, Carraher MJ, Bennett PH, Knowler WC (1988). Congenital susceptibility to NIDDM. Role of intrauterine environment. Diabetes.

[32] Stride A, Shepherd M, Frayling TM, Bulman MP, Ellard S, Hattersley AT (2002). Intrauterine hyperglycemia is associated with an earlier diagnosis of diabetes in HNF-1a gene mutation carriers. Diabetes Care.

[33] Sobngwi E, Boudou P, Mauvais-Jarvis F, Leblanc H, Velho G, Vexiau P (2003). Effect of a diabetic environment in utero on predisposition to type 2 diabetes. Lancet.

[34] Prokopenko I, Langenberg C, Florez JC, Saxena R, Soranzo N, Thorleifsson G (2009). Variants in MTNR1B influence fasting glucose levels. Nat Genet.

[35] Freathy RM, Weedon MN, Bennett A, Hypponen E, Relton CL, Knight B (2007). Type 2 diabetes TCF7L2 risk genotypes alter birth weight: a study of 24,053 individuals. Am J Hum Genet.

[36] Weedon MN, Clark VJ, Qian Y, Ben-Shlomo Y, Timpson N, Ebrahim S (2006). A common haplotype of the glucokinase gene alters fasting glucose and birth weight: association in six studies and population-genetics analyses. Am J Hum Genet.

[37] Freathy RM, Hayes MG, Urbanek M, Lowe LP, Lee H, Ackerman C (2010). Hyperglycemia and adverse pregnancy outcome (HAPO) study: common genetic variants in GCK and TCF7L2 are associated with fasting and postchallenge glucose levels in pregnancy and with the new consensus definition of gestational diabetes mellitus from the International Association of Diabetes and Pregnancy Study Groups. Diabetes.

[38] Dimas AS, Lagou V, Barker A, Knowles JW, Mägi R, Hivert MF (2014). Impact of type 2 diabetes susceptibility variants on quantitative glycemic traits reveals mechanistic heterogeneity. Diabetes.

[39] Zhang G, Bacelis J, Lengyel C, Teramo K, Hallman M, Helgeland Ø (2015). Assessing the causal relationship of maternal height on birth size and gestational age at birth: a Mendelian randomization analysis. PLoS Med.

[40] Kong A, Steinthorsdottir V, Masson G, Thorleifsson G, Sulem P, Besenbacher S (2009). Parental origin of sequence variants associated with complex diseases. Nature.

